# DNA barcoding of aphid-associated ants (Hymenoptera, Formicidae) in a subtropical area of southern China

**DOI:** 10.3897/zookeys.879.29705

**Published:** 2019-10-09

**Authors:** Junaid Ali Siddiqui, Zhilin Chen, Qiang Li, Jun Deng, Xiaolan Lin, Xiaolei Huang

**Affiliations:** 1 State Key Laboratory of Ecological Pest Control for Fujian and Taiwan Crops, College of Plant Protection, Fujian Agriculture and Forestry University, Fuzhou 350002, China; 2 Fujian Provincial Key Laboratory of Insect Ecology, Fujian Agriculture and Forestry University, Fuzhou 350002, China; 3 Key Laboratory of Ecology of Rare and Endangered Species and Environmental Protection of Ministry of Education, Guangxi Normal University, Guilin 541004, China

**Keywords:** cryptic diversity, DNA barcode, genetic distance, myrmecophily

## Abstract

As one of the most abundant and complex groups of terrestrial insects, ants have associations with many other organismal groups, such as hemipteran insects producing honeydew. With the aim of expanding the knowledge base of ant species associated with aphids, this study analyzed mitochondrial COI barcodes of 301 ant samples for 37 aphid-associated ant species in a subtropical area of southern China. Sequence analyses revealed that the intraspecific and interspecific distances ranged from zero to 7.7%% and 0.2 to 31.7%, respectively. Three barcoding approaches – Automatic Barcode Gap Discovery, Bayesian Poisson Tree Processes and Generalized Mixed Yule-coalescent – were used to help delimit ant species based on COI sequences, and their results corresponded well with most of the morphospecies. All three approaches indicate cryptic diversity may exist within *Tetramorium bicarinatum* and *Technomyrmex albipes*, with intraspecific genetic distances of 7.7% and 6.24%, respectively. Our analyses also reported five species for the first time from Fujian Province of China, and the COI sequences of nine species are newly added into the GenBank. This study provides information about species diversity of aphid-associated ants in subtropical China and compiles a DNA barcode reference library for future ant barcoding work.

## Introduction

Relationships between various organisms are crucial for upholding the ecological function of natural communities. The interactions between ants and aphids are classic examples of mutualism and are important to support ecosystem function (Fischer et al. 2015). The aphids have positive interactions with ants, which can play important role in their survival rate. The ant-aphid associations even have a great impact on local insect faunal diversity (Billick et al. 2007), especially dominant ants. They even determine the structure of the local ant community by interfering with the foraging of other ants (Carval et al. 2016). These interactions are very diverse and complex in nature. For a better understanding of their complex interactions, it is very important to know the species diversity of each part of this association. Ants are the part that collect honeydew of aphids and protect them from natural enemies (Stadler and Dixon 2005). The diversity of ants is highest in tropical regions, while aphids are supposed to be most diverse in temperate regions (Hölldobler and Wilson 1990, Heie 1994). As transition zones where the two groups encounter one another, subtropical regions may have an elevated diversity of ant-aphid associations. However, there have been no published studies focusing on diversity of both partners in this association. In this work, we tried to explore the diversity of aphid-associated ants in the subtropical Fujian in southern China.

Ants (Hymenoptera, Formicidae) are a dominant terrestrial insect group. They have colonized almost the entire world except Antarctica, especially in the tropical regions (Rizali et al. 2008). There are 17 subfamilies having about 13,500 described species worldwide (Bolton 2018). This group of insects has been present on Earth for about 120 Mya (Brady 2003). Ants play essential roles in seed dispersal (Hanzawa et al. 1988). Many grass species in fire-prone graze lands mainly depend on ants for their survival (Fisher et al. 2003). Also, they are efficient biocontrol agents and improve soil aeration as well (Hölldobler and Wilson 1990). For example, the predatory Asian weaver ants are the most efficient biocontrol agents of certain field crops and predatory ants of genus *Oecophylla* can control 50 different species of pests species feeding on eight tropical trees (Peng and Christian 2009, Offenberg 2014). Due to the obligatory interactions between ants, plants and other animals, the diversity of ants usually is a good indicator of the strength of ecosystems (Alonso and Agosti 2000).

Biological classification based on morphological characters has been a routine practice to identify biodiversity on the Earth. Nowadays, biodiversity quantification is a challenge for taxonomist if only based on morphological identification. The recognition of minute anatomical differences between closely related species sometimes is complicated morphologically (Ojha et al. 2014). Ants usually have different castes with apparent variations in their body structure within the same species, which makes them more diverse and challenging to identify. To overcome these problems, DNA barcoding has been shown to be a reliable technique for rapid and accurate species identification (Hebert et al. 2003a, Savolainen et al. 2005). Mitochondrial DNA (mtDNA) has been extensively used in molecular studies. A partial fragment of cytochrome c oxidase I gene (COI) is employed for easy identification of closely related or cryptic animal species along with biological diversity assessment (Hebert et al. 2004, Ojha et al. 2014). The utility of DNA barcoding as a rapid and accurate tool for species identification is well recognized in a wide variety of animal taxa across the globe (http://www.ibol.org/resources/). DNA barcoding techniques have been used by some researchers in ant identification and phylogenetic analysis (Smith et al. 2005, Jansen et al. 2009, Ng’endo et al. 2013, Smith et al. 2013, Ojha et al. 2014, Chen and Zhou 2017). However, to our knowledge, little is known about the regional fauna of aphid-associated ants especially in subtropical areas.

The present study aimed to investigate the subtropical ant fauna associated with aphids with the help of DNA barcoding. Both the morphological and DNA barcoding approaches were used and results were compared. Our study provides information of species composition and species diversity of ants in a subtropical region, and a DNA library for future ant barcoding work.

## Materials and methods

### Sample collection

**Figure 1.**
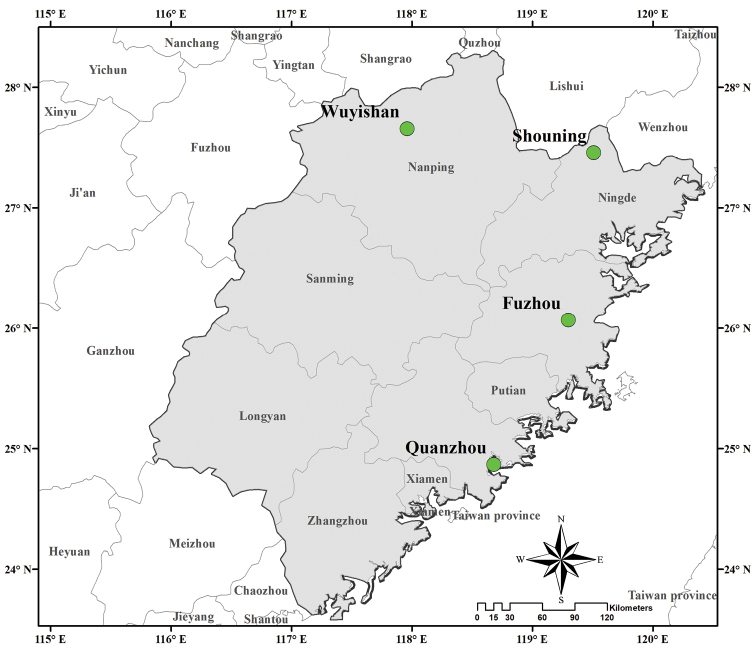
Map of Fujian Province showing the sampling sites.

The ant specimens were collected from four localities (Fuzhou, Quanzhou, Shouning, Wuyishan) (Fig. 1) of the subtropical areas of Fujian Province in southern China. Specimens were collected during 2015–2017 by hand and camel hair brushes. Ant collection was based on the occurrence of aphids in different places. During our field collections, on the basis of visual observations, only the ant individuals attending aphid populations with obvious physical contact of beating aphid body by antenna and a consistent feeding on aphid honeydew were collected. All collected specimens were placed in 95% alcohol and kept in -20 °C until processed. Ant samples were identified morphologically first. The complete dataset comprises 301 individual specimens representing three subfamilies, 19 genera and 37 species (Suppl. material 2: Table S2).

### Morphological identification

The ant species were identified by Dr Chen Zhilin and Dr Zhou Shanyi (Guangxi Normal University, Guilin 541004, China). Both of them have described more than 100 ant species up till now. Their knowledge and expertise help guarantee the reliability of the morphological identification. The voucher specimens have been stored at the Insect Systematics and Diversity lab at Fujian Agriculture and Forestry University.

### DNA extraction and PCR amplification and sequencing

DNA was isolated from the leg or whole ant body using the Qiagen DNeasy kit following the manufacturer’s protocols. Mainly a non-destructive DNA extraction method was used. In cases where numerous individuals from a colony were available, a destructive technique (entire ant crushed) was preferred. Polymerase chain reactions (PCR) were carried out in a total reaction volume of 50 µL containing 8 µL of dNTP mixture (2.5 mM), 5 µL of 10× PCR buffer (25 mM Mg^2^), 10 pmol of each primer and I unit of Taq DNA polymerase (TaKaRa Bio Inc., Otsu, Japan). The reaction conditions for the COI gene include: initial denaturation at 95 °C for 5 min; 35 cycles of 94 °C for 1 min, 50 °C for 1 min (denaturing) and 72 °C for 1 min (extension); a final elongation at 72 °C for 7 min reactions were done using the ProFlex PCR system. Standard primers used were: forward primer LepF1 (ATTCAACCAATCATAAAGATATTGG) and reverse primer LepR1 (TAAACTTCTGGATGTCCAAAAAATCA) (Hebert et al. 2003b). The amplified products were visualized on 1% agarose gel stained with ethidium bromide. PCR purified products were sent to a (Sangon Biotech (Shanghai) Co., Ltd) for bidirectional sequencing. Obtained sequences were manually edited in BioEdit version 7.0.5.0 (Hall 1999) and aligned with MAFFT v7 (Katoh et al. 2009). The resultant sequence fragments were around 600–700 base pairs (bp). These sequences were identified as COI fragments for the ants with BLAST procedure searched in public database (Altschul et al. 1990). The aligned sequences were 593 bp long and free from gaps after trimming. All sequences were deposited in the GenBank under accession number (MH754200-MH754506) and BOLD under process IDs (DBAFC001-19-DBAFC301-19).

### Sequence analysis and species delimitation

A total of 301 sequences from our study (Suppl. material 2: Table S2) and 52 COI sequences (mostly sequences with BLAST results) collected from GenBank were included in further analyses. Moreover, *Vespula germanica* (KR788643.1) and *Vespa velutina* (LC170010.1) were used as outgroups. Pairwise intraspecific genetic distance was calculated between all sequences of same species, while pairwise interspecific distance between species of the same genera and all species of a subfamily under Kimura-2-Parameter (K2P) and Proportional (p-distance) distances models were calculated using MEGA 7.0 (Kumar et al. 2016). The sequences were without stop codons, frameshift mutations or a high dN/dS ratio, which helped us to conclude that they were mitochondrial and not nuclear mitochondrial DNA segment (NUMTs) (Bensasson et al. 2001, Calvignac et al. 2011). Analyzing the unidentified NUMTs as the true mitochondrial sequences could result in the inappropriate identification of cryptic species (Song et al. 2008). Based on our analysis performed, we are confident that the sequences analyzed here are mitochondrial in origin.

Automatic Barcode Gap Discovery (ABGD) (Puillandre et al. 2012), Bayesian Poisson Tree Processes (bPTP) (Zhang et al. 2013) and Generalized Mixed Yule-coalescent (GMYC) (Pons et al. 2006) were used for species delimitation. The ABGD method was performed for detecting the barcode gaps and identification of distinct clusters of COI sequences. The maximum value of intraspecific divergence was fixed between 0.001 and 0.1. Moreover, the K2P model (Kimura 1980) was used along with the default gap width of X=1.5. In the PTP analysis, distinctive haplotype sequences were obtained using DnaSP 6.10 (Librado and Rozas 2009), then phylogenetic trees were constructed based on these haplotype sequences by using raxmlGUI v1.5 (Stamatakis 2014). The GTR+I+G model was the best model obtained by jModel test v2.1.7 (Posada 2008). This method is implemented in an online web server (http://species.h-its.org/). For the GMYC model, firstly a linearized Bayesian phylogenetic tree was calculated in BEAST v1.8.4 using a Yule pure birth model tree prior. Settings in BEAUTi v1.8.4 were: best substitution model, estimated base frequencies, four gamma categories. An uncorrelated relaxed log-normal clock model was used with a log-normal relaxed distribution. All further settings were left as defaults. The Markov Chain Monte Carlo length was 100,000,000 generations with log parameters every 10,000 generations. The evaluation of ESS values and trace files of runs were performed in Tracer v1.6. Tree files obtained from BEAST analysis were combined using the LogCombiner prior to generating the final ultra-metric tree with 20% burn-in, 0.5 posterior probability limit, and node heights of target tree were performed in TreeAnnotator v1.8.4. Single-threshold GMYC analyses were carried out in R studio using the PARAN, APE and SPLITS packages.

The maximum likelihood (ML) (Tamura et al. 2011) and Bayesian approaches (Huelsenbeck and Ronquist 2001) were also used to build phylogenetic trees. The ML tree was constructed based on haplotype sequences by using raxmlGUI v1.5 (Stamatakis 2014). The best nucleotide substitution model for the COI sequences for ML analysis was selected on the basis of the Bayesian information criterion value by jModeltest v2.1.7 (Posada 2008). The most suitable model for ML analysis was GTR+I+G for haplotypes among the 301 sequences identified by DnaSP 6.10 (Librado and Rozas 2009).

A BI tree was reconstructed under the GTR+I+G (Bollback 2002) model (obtained by jModel Test) for all sequence of current study and combined with GenBank sequences in MrBayes v3.2.6 (Ronquist et al. 2012) with two independent runs and each run employing four Metropolis Coupled Monte Carlo Markov chains (three heated and one cold). The number of generations for the total analysis was set at 100 million. The burn-in value was set as 25% and other parameters were left as default options. The evaluating effective sample size values were analyzed in Tracer v1.6 (Rambaut et al. 2014), and generated trees were visualized in FigTree v1.4.3 (http://tree.bio.ed.ac.uk/software/figtree) and edited in MEGA 7.0 (Kumar et al. 2016).

## Results

A total of 37 ant species associated with aphids were identified morphologically, belonging to 19 genera of three subfamilies, viz., Dolichoderinae (8), Formicinae (16) and Myrmicinae (13) (Suppl. material 2: Table S2). COI sequences were obtained from all the 301 samples used. The newly acquired 301 COI sequences were deposited in GenBank and BOLD. BLAST analysis in the NCBI database showed overall 84–100% nucleotide identity between the newly acquired sequences and the previously published COI sequences in GenBank. In this study, the COI sequences of nine species, namely *Aphaenogaster smythiesii*, *Crematogaster nicobarensis*, *C. vitiosus*, *C. egidyi*, *C. osakensis*, *Monomorium chinense*, *Pheidole fervida*, *P. smythiesii* and *Nylanderia flaviabdominis* were newly added in GenBank. Our results also found five species, namely *Formica sinae*, *N. flaviabdominis*, *Prenolepis emmae*, *C. egidyi*, and *Pheidole smythiesii* that were newly recorded from Fujian Province of China.

The specimens collected from the Wuyishan Nature Reserve showed maximum species diversity up to 21 species, whereas the other two localities, Shouning and Fuzhou, had almost similar species diversity with 18 and 16 species respectively. The subfamily Myrmicinae had highest number of taxa in our study, with seven genera and 13 species occupying 55.48% of the total 301 samples. The genus *Crematogaster* was the most dominate group representing 23% of total samples. Moreover, three ant species *P. punctatus*, *C. egidyi* and *P. noda* showed the mostaphid associations with 17, 16 and 12 aphid species respectively (Suppl. material 1: Table S1).

### Genetic distances

Intraspecific divergences were calculated for all species except those with only one sequence while interspecific distances were calculated for subfamilies and genera using p-distance and K2P model (Table 1). Moreover, we found that the values of the genetic distance calculated by the K2P model were slightly higher than the p-distance. The results of both models (p-distance and K2P model) were similar (Suppl. material 3: Figure S1), so for further analysis the K2P distance only was used. The intraspecific distances for most species were lower than 1%, the maximum intraspecific distance varied from 1.3% (e.g., *N. flaviabdominis*) to 7.7% (*Tetramorium bicarinatum)*, and the mean intraspecific distances varied from 0.01 (*Nylanderia flavipes*) to 0.043 (*Tetramorium bicarinatum*). The maximum interspecific distances for the three subfamilies were: Myrmicinae 31.7%, Formicinae 31.6% and Dolichoderinae 27%. For some abundant genera based on sample numbers, the interspecific distances were: *Camponotus* 0.195–0.251, *Crematogaster* 0.056–0.229, *Nylanderia* 0.139–0.222, *Tetramorium* 0.169–0.218, *Pheidole* 0.167–0.199, and *Formica* 0.002–0.005. Overall interspecific distance of the 301 COI sequences ranged from 0.048 to 0.345 (Table 1).

**Table 1.** COIK2P genetic distances for aphid-associated ant species in this study. Intraspecific distances were calculated within the same species and interspecific distances between species of same genus. Only species with two or more sequences were included.

Taxon name	Number of sequences	Intraspecific distance			Interspecific distance	Number of haplotypes
		min.	max.	mean	range	
** Dolichoderinae **					0.137–0.27	
*Iridomyrmex anceps*	7	0	0	0		1
*Liometopum sinense*	2	0	0	0		1
*Ochetellus glaber*	6	0	0.005	0.002		2
*Tapinoma melanocephalum*	9	0	0.034	0.008	0.139–0.258	2
*Technomyrmex albipes*	8	0	0.062	0.033		2
** Formicinae **					0.048–0.316	
*Camponotus japonicus*	9	0	0.007	0.003	0.195–0.251	5
*Camponotus mitis*	2		0.008	0.008	0.195–0.251	2
*Camponotus nicobarensis*	5	0	0.002	0.001	0.195–0.251	2
*Formica japonica*	14	0	0	0	0.002–0.005	1
*Formica sinae*	12	0	0.005	0.002	0.002–0.005	6
*Lepisiota rothneyi*	2	0	0	0	0.002–0.005	1
*Nylanderia flavipes*	10	0	0.022	0.010	0.139–0.222	2
*Nylanderia bourbonica*	11	0	0.002	0.001	0.139–0.222	2
*Nylanderia flaviabdominis*	6	0	0.013	0.007	0.139–0.222	2
*Polyrhachis dives*	13	0	0.015	0.006	0.175–0.184	3
*Polyrhachis illaudata*	3	0	0.002	0.001	0.175–0.184	2
*Prenolepis emmae*	6	0	0.003	0.001		3
** Myrmicinae **					0.056–0.317	
*Aphaenogaster smythiesii*	2	0	0	0		1
*Crematogaster egidyi*	53	0	0.015	0.007	0.056–0.229	6
*Crematogaster osakensis*	5	0	0.003	0.001	0.056–0.229	3
*Crematogaster rogenhoferi*	10	0	0.005	0.002	0.056–0.229	2
*Monomorium chinense*	3	0	0	0	0.175	1
*Pheidole fervida*	2	0	0	0	0.167–0.199	1
*Pheidole noda*	30	0	0.020	0.009	0.167–0.199	5
*Pristomyrmex punctatus*	46	0	0.058	0.016		8
*Tetramorium wroughtonii*	2	0	0	0	0.169–0.218	1
*Tetramorium bicarinatum*	9	0	0.077	0.043	0.169–0.218	2
*Tetramorium caespitum*	3	0	0.020	0.013	0.169–0.218	2
**Total**					0.048–0.345	

### Species delimitations

**Figure 2.**
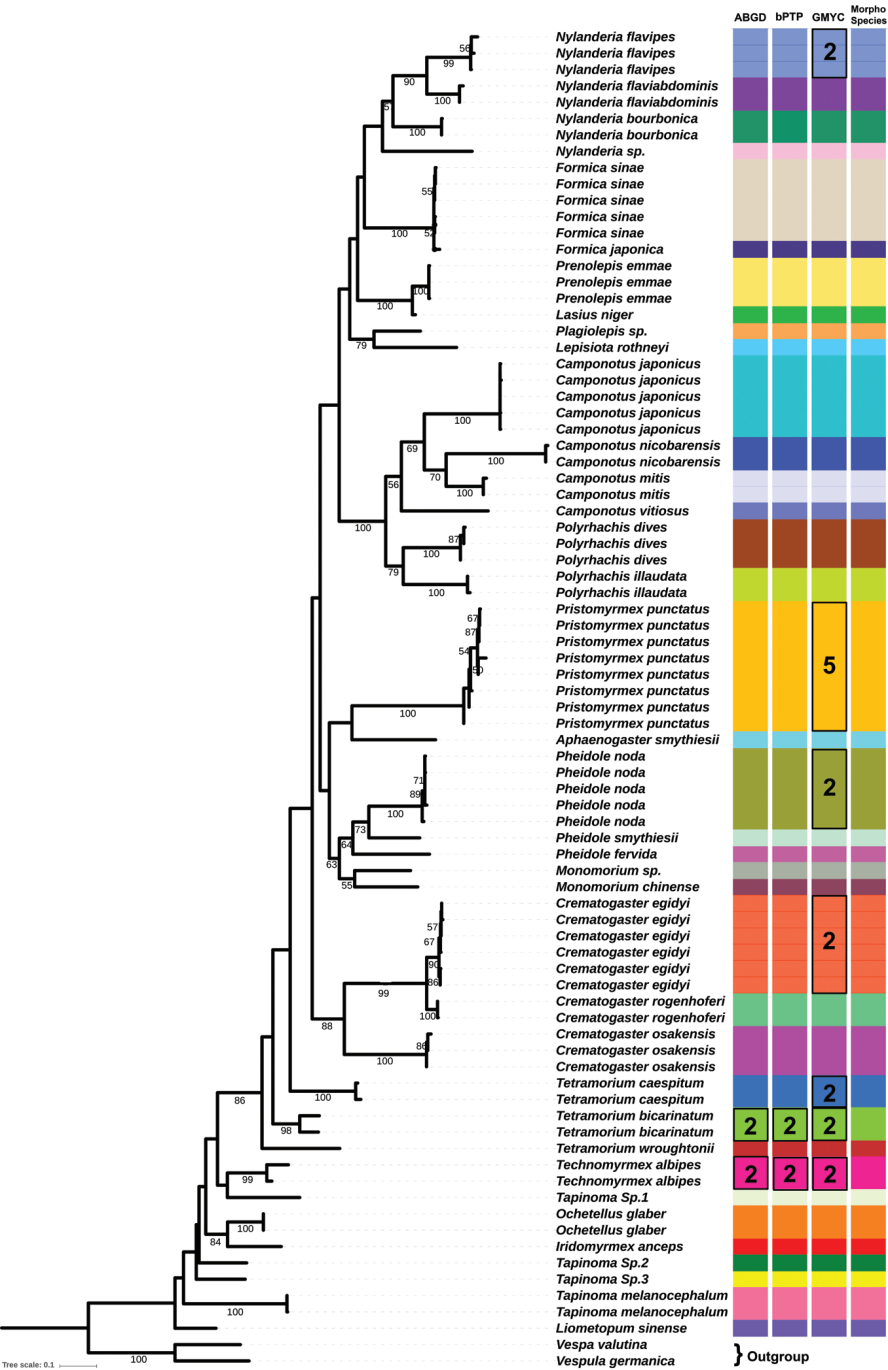
Maximum likelihood haplotype tree for the COI gene. Bootstrap values higher than 50 are displayed. Color strips on the right side represent the MOTUs produced by ABGD, bPTP and GMYC methods; extreme right one indicates the morphologically identified species. Black square around some bars indicates differences between the MOTUs and morphospecies. Values inside the square indicate the number of MOTUs produced by different approaches.

The ABGD approach produced 39 molecular operational taxonomic units (MOTUs) or genetic groups. Among them, 35 MOTUs matched with the morphospecies identification, which represented 89.7% of the morphospecies in total. The other four MOTUs might indicate species differentiation for some morphospecies (Fig. 2). These included *T. caespitum* and *T. albipes*, which were each divided into two groups. The bPTP approach yielded the same species delimitation result as the ABGD, also dividing the 301 sequences into 39 putative species and 35 of them corresponding well to morphological identifications. The GMYC method produced different results: the 301 sequences were grouped into 47 MOTUs; 30 MOTUs were congruent with the ABGD and bPTP as well as the morphospecies (Fig. 2). Contrary to the other two approaches, *P. punctatus* was divided into five separate MOTUs. Moreover, species *C. egidyi*, *N. flavipes*, *P. noda*, *T. caespitum*, *T. albipes*, *T. bicarinatum* were each separated into two groups.

**Figure 3.**
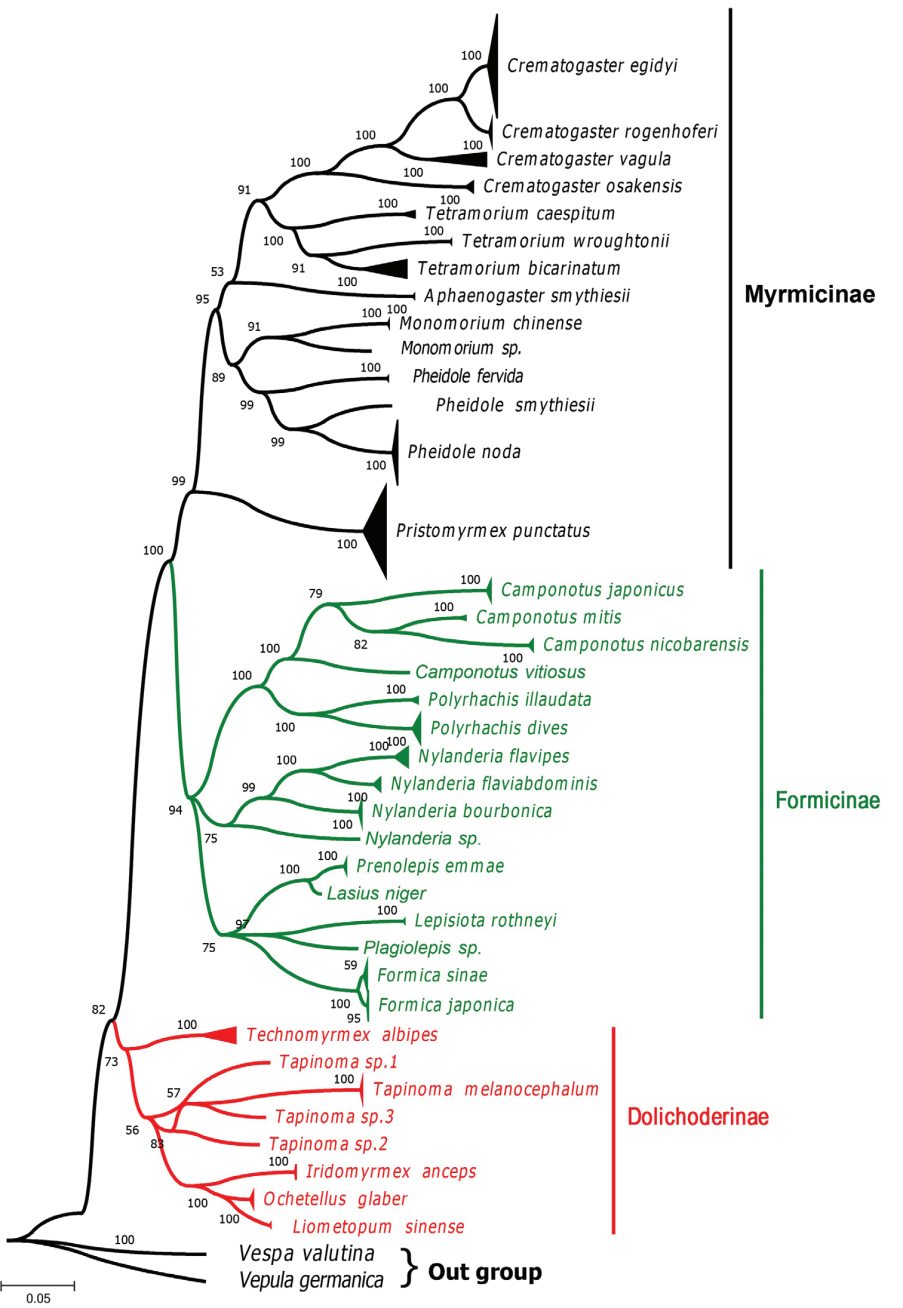
Bayesian inference tree for the COI gene. The numbers on the branches are Bayesian posterior probabilities. The black, green and red colours indicate the species under each subfamily, respectively.

**Figure 4.**
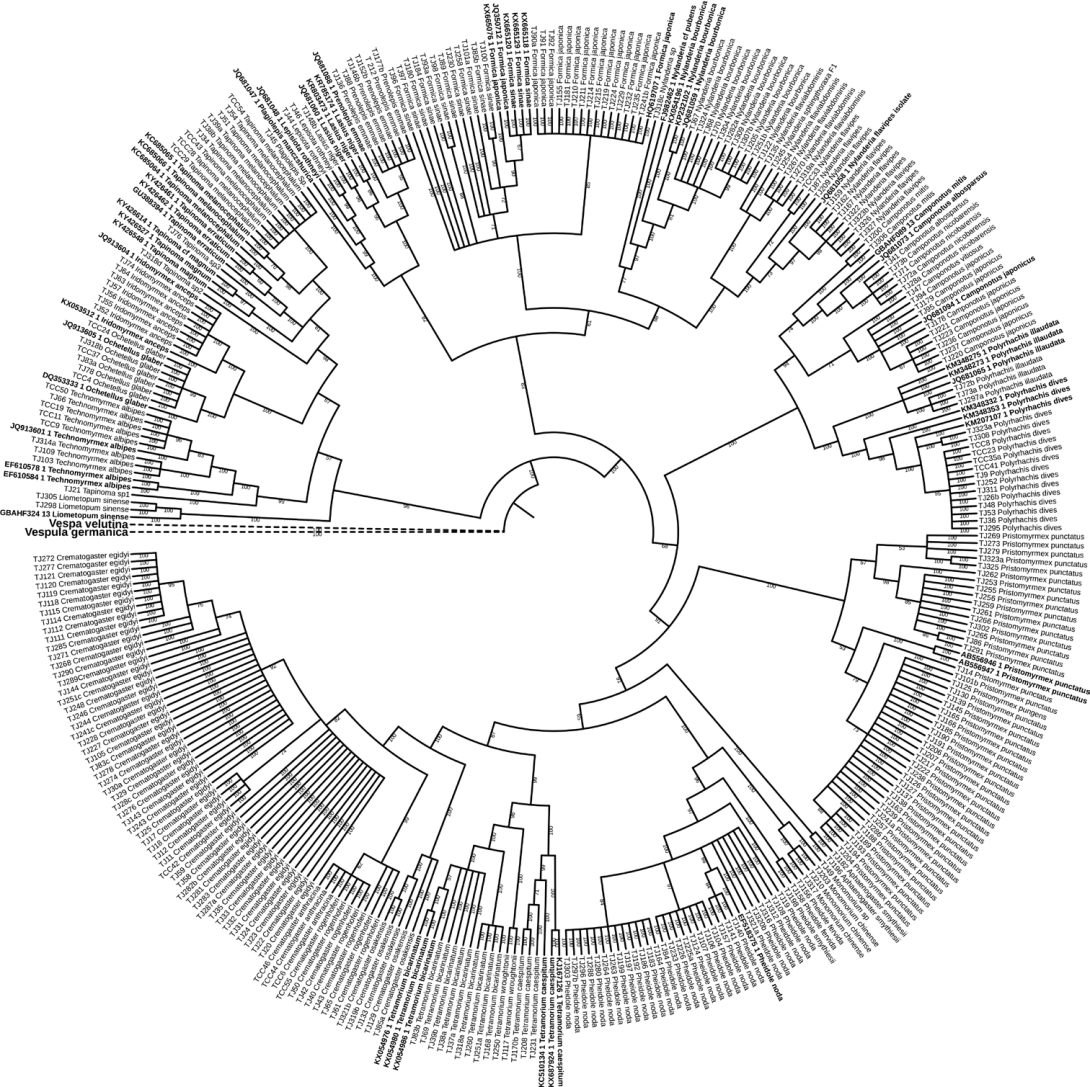
Bayesian Inference tree combined with 301 COI sequences from the current study and 52 COI sequences from the GenBank. Values besides the branches indicate Bayesian posterior probabilities. Dotted lines are indicating the outgroups. Bold labels indicate the sequences from the GenBank.

ML and Bayesian Inference analysis applied to all 303 sequences along with two outgroups created monophyletic groups. As the phylogenetic trees (Figs 2–4, Suppl. material 4: Figure S2) show, all the 37 morphospecies were clustered into three subfamilies, Dolichoderinae, Formicinae and Myrmicinae. The relationships between these three subfamilies revealed in our study were similar to those reported in previous studies (Kück et al. 2011, Reemer 2013). Different clades or groups in the phylogenetic trees corresponded well with the MOTUs produced by the ABGD, bPTP, and GMYC methods.

## Discussion

Ants are eusocial insects having the high degree of caste polymorphism with various distinct anatomical characters and size variations (Wheeler 1986, Mysore et al. 2009, Wills et al. 2018). The complexity of ant groups usually makes them difficult to identify to species only based on morphological characters. For example, species of genus *Crematogaster* have been reported to be morphologically diverse and having cryptic species with high genetic intraspecific variation (Blaimer 2012). There are few studies that combine morphological identification with DNA barcode analysis for ants (Ng’endo et al. 2013, Kanturski et al. 2018). However, various barcoding approaches of species delimitation can be more suitable and useful in describing ant species diversity (Smith et al. 2005, Ojha et al. 2014). Our paper may be the first study of a regional fauna of aphid-associated ants to use a combined species delimitation approach.

Most of the morphospecies identified were supported by DNA barcoding approaches. According to the Guénard and Dunn (2012) Fujian Province has 184 ant species and this study added five new ant species (*F. sinae*, *N. flaviabdominis*, *P. emmae*, *C. egidyi* and *P. smythiesii*) to the provincial ant fauna. Moreover, COI sequences of nine species (*C. nicobarensis*, *C. vitiosus*, *A. smythiesii*, *C. egidyi*, *C. osakensis*, *M. chinense*, *N. flaviabdominis P. fervida* and *P. smythiesii*) were newly added into the GenBank and BOLD databases. Geographically, we also found highest species diversity in the Wuyishan Nature Reserve with 21 species. This is related to the fact that the Wuyishan Nature Reserve has the highest habit heterogeneity compared to the other three geographic areas (Ding et al. 2015).

In the present study, three ant species (*P. punctatus*, *C. egidyi* and *P. noda*) were found associated with a maximum number of aphid species on various host plants (Suppl. material 1: Table S1). All of them belong to the subfamily Myrmicinae. The parthenogenetic ant species *P. punctatus* is known as seed harvester ants; they are abundantly present in forests and natural vegetation (Satow et al. 2013; Zhu and Wang 2018). They have the ability to fuse their colony into neighbouring colonies of same species (Satow et al. 2013), which may make them more abundant. In a previous study *P. punctatus* was found to be the most dominant ant in natural grassland in Japan (Suetsugu 2015). In the current study this species is mainly found in natural vegetation in Shouning and Wuyishan Nature Reserve interacting with 17 aphid species. Due to their high abundance, this species was found as the most dominant aphid-associated ant in our study areas. *Crematogaster egidyi* is known as an aggressive predatory arboreal ant species (Longino 2003). They are considered to be strong and aggressive towards other dominant ant species and compete for food and space (Richard et al. 2001). In the current study *C. egidyi* was found associated with 16 species of aphids. In our sampling sites this species was also found dominant and aggressive towards other ant foragers, which may influence the local ant diversity. *Pheidole noda* is a seed dispersal ant species mostly found in the open lands and forest vegetation (Yamawo et al. 2012) and mainly distributed in the east Asian countries (Sarnat et al. 2015).It has been found abundant from Iwo-jima island of Japan (Ikudome and Yamane 2007) and also reported from rainforest of Yuanan, China (Liu et al. 2015). In the current study, *P. noda* was also found abundantly associated with 12 aphid species on various host plants in the Wuyishan Nature Reserve and mountainous areas of Shouning. It was observed that these three species associated with the most aphids, mainly found in natural habitats, and therefore may influence other aphid-associated ant species.

Species delineation and identification on the basis of DNA sequence distance analysis, like the DNA barcoding gap (Hebert et al. 2003b, Hebert et al. 2004) and other related methodologies (Ferri et al. 2009), have been used repeatedly to develop effective standards for species delimitation. Genetic distance-based methods are regularly being used in DNA barcoding studies of various groups to indicate the possible incidence of cryptic species diversity among morphologically similar species (Lefébure et al. 2006), including termites (Roy et al. 2014), butterflies (Ashfaq et al. 2013) and snails (Prévot et al. 2013). Genetic divergence of ant species was previously calculated by different researchers on the basis of COI gene sequences, but they focused on ant groups solely (Ng’endo 2011, Ojha et al. 2014). Actually, the interactions between ants and other insect groups, for example aphids, are critical to regional community function. Considering that morphologically similar ant species may occur in a same area and sometimes co-occur with same aphid species, molecular identification is helpful to understand the regional diversity of aphid-associated ants. In this study, we observed that several morphospecies produced multiple MOTUs using the different barcoding methods; also, the MOTUs were separated in the phylogenetic tree analysis (Figs 2–4, Suppl. material 4: Figure S2). The ABGD, bPTP and GMYC methods all supported *T. albipes* and *T. bicarinatum* as each having two clear MOTUs. These two species showed higher mean (>3.34%) as well as maximum (>6.24%) intraspecific genetic distances. The GMYC method revealed five more species with multiple MOTUs: *P. punctatus*, *N. flavipes*, *P. noda*, *T. caespitum* and *C. egidyi*. The maximum intraspecific distances were 5.84%, 2.19%, 2.01%, 2.01% and 1.51% respectively; however, the mean intraspecific genetic distances were all below 1.55%, which is lower than the practical criterion for insect species delimitation (Foottit et al. 2008). This may be the reason that the ABGD and bPTP methods found them each to be a single MOTUs.

The comparative performance of different algorithms to species delineation has been studied previously. ABGD considered as the most computationally effective approach. It needs a priori specification of an intraspecific distance threshold, and this method is based on the genetic distances calculated from a single locus (Puillandre et al. 2012). Empirical studies have revealed that the GMYC approach tends to over-split species compared to alternative methods of species delimitation (Esselstyn et al. 2012, Paz and Crawford 2012, Sauer and Hausdorf 2012, Talavera et al. 2013). Other studies indicate that the ABGD and bPTP may be better strategies and that these encounter less computing errors than GMYC (Puillandre et al. 2012, Luo et al. 2018). Our study also showed GMYC delimited more MOTUs. However, considering GMYC combines the Yule model of species birth with neutral coalescent model of intraspecific branching, its results may also have implications for understanding population divergence for some species. For example, previous studies reported diverse cryptic species within the *T. caespitum* complex (Wagner et al. 2018). In this study, the individuals of *T. caespitum* were grouped into two MOTUs by GMYC, and the maximum intraspecific genetic distance was 2.0%, which may also indicate possible differentiation within this species.

The ML and BI phylogenetic analysis produced almost same topologies on the basis of the COI sequences and produced two discrete clades. One clade included two putative sister clades representing the subfamilies Myrmicinae and Formicinae (Figs 3, 4). It has been thought that the two subfamilies evolved from a common ancestor (Brady et al. 2006, LaPolla et al. 2010, Ward et al. 2015). Moreover, a second cluster comprised species of subfamily Dolichoderinae. The grouping of the six genera presented in this study was similar to that reported by Chiotis et al. (2000), but our DNA sequence data alone provide insufficient support to comment on relationships among the ant subfamilies. Leaving aside the assemblages supported by lower posterior probability values, on the basis of the sole barcode sequence data set, the three subfamilies (Dolichoderinae, Formicinae, and Myrmicinae) mostly appear as monophyletic. The overall topology of phylogenetic trees corresponds well with most results of ABGD, bPTP and GMYC species delimitation approaches. For the new MOTUs we found beyond the known morphospecies further DNA diagnostics based on more sampling and morphological work are needed to verify whether they can be well-defined species.

## Supplementary materials

Supplementary material 1 Table S1. Ant and associated aphid species collected from different localities of Fujian Province of southern China Data type: species data https://binary.pensoft.net/file/345492This dataset is made available under the Open Database License (http://opendatacommons.org/licenses/odbl/1.0/). The Open Database License (ODbL) is a license agreement intended to allow users to freely share, modify, and use this Dataset while maintaining this same freedom for others, provided that the original source and author(s) are credited.Junaid Ali Siddiqui, Zhilin Chen, Qiang Li, Jun Deng, Xiaolan Lin, Xiaolei Huang

Supplementary material 2 Table S2. Analyzed samples of ant species with information on sampling location, GenBank accession number, BOLD process ID, morphological species identification, collection date, voucher specimen number, latitude and longitude Data type: species data https://binary.pensoft.net/file/345493This dataset is made available under the Open Database License (http://opendatacommons.org/licenses/odbl/1.0/). The Open Database License (ODbL) is a license agreement intended to allow users to freely share, modify, and use this Dataset while maintaining this same freedom for others, provided that the original source and author(s) are credited.Junaid Ali Siddiqui, Zhilin Chen, Qiang Li, Jun Deng, Xiaolan Lin, Xiaolei Huang

Supplementary material 3 Figure S1. Line chart of genetic distance of different taxonomic level based on p-distance and K2P model Data type: molecular data https://binary.pensoft.net/file/345494This dataset is made available under the Open Database License (http://opendatacommons.org/licenses/odbl/1.0/). The Open Database License (ODbL) is a license agreement intended to allow users to freely share, modify, and use this Dataset while maintaining this same freedom for others, provided that the original source and author(s) are credited.Junaid Ali Siddiqui, Zhilin Chen, Qiang Li, Jun Deng, Xiaolan Lin, Xiaolei Huang

Supplementary material 4 Figure S2. Bayesian Inference tree of the 301 COI sequences from our study Data type: phylogenetic tree Explanation note: The values besides the branches indicate Bayesian posterior probabilities. The dotted lines indicate the outgroups. https://binary.pensoft.net/file/345495This dataset is made available under the Open Database License (http://opendatacommons.org/licenses/odbl/1.0/). The Open Database License (ODbL) is a license agreement intended to allow users to freely share, modify, and use this Dataset while maintaining this same freedom for others, provided that the original source and author(s) are credited.Junaid Ali Siddiqui, Zhilin Chen, Qiang Li, Jun Deng, Xiaolan Lin, Xiaolei Huang
