# Supplementary material for: DNA barcoding of aphid-associated ants (Hymenoptera, Formicidae) in a subtropical area of southern China
Source: Zookeys. 2019 Oct 9;879:117–36. doi: 10.3897/zookeys.879.29705 (PMC6795625; doi:10.3897/zookeys.879.29705)
Supplement: Supplementary material 1 [file zookeys-879-117-s001.docx]

Supplementary Table 1. Ant and associated aphid species collected from different localities of Fujian province of southern China.

| **Ant species** | **Aphid species** | **Localities** |
| --- | --- | --- |
| *Aphaenogaster smythiesii* | *Aphis gossypii, Semiaphis heraclei* | Wuyishan |
| *Camponotus japonicus* | *Aphis spiraecola, Cinara formosana, Trichosiphonaphis lonicerae, Uroleucon erigeronensis* | Wuyishan |
| *Camponotus mitis* | *Aphis spiraecola* | Shouning, Wuyishan |
| *Camponotus nicobarensis* | *Aphis aurantii, Pseudoregma bambucicola,* | Fuzhou |
| *Camponotus vitiosus* | *Greenidea ficicola* | Quanzhou |
| *Crematogaster egidyi* | *Aphis aurantii, Aphis craccivora, Aphis gossypii, Aphis rumicis, Aphis spiraecola, Aphis odinae, Astegopteryx bambucifoliae, Chaitoregma tattakana, Eutrichosiphum manipurense, Kurisakia onigurumii, Matsumuraja rubifoliae, Melanaphis japonica, Mollitrichosiphum tenuicorpus, Ovatus crataegarius, Periphyllus koelreuteriae, Pseudoregma bambucicola* | Fuzhou, Shouning, Wuyishan |
| *Crematogaster osakensis* | *Aphis fabae solanella, Aphis gossypii, Aphis glycines* | Shouning, Wuyishan |
| *Crematogaster rogenhoferi* | *Aphis aurantii, Aphis gossypii, Aphis odinae, Greenidea psidii, Mollitrichosiphum tenuicorpus* | Fuzhou |
| *Formica japonica* | *Aphis aurantii, Aphis odinae, Aphis gossypii, Aphis spiraecola, Matsumuraja rubifoliae, Megoura lespedezae, Semiaphis heraclei, Trichosiphonaphis lonicerae* | Wuyishan |
| *Formica sinae* | *Aphis glycines, Aphis gossypii, Aphis spiraecola, Betulaphis pelei, Ceratovacuna hoffmanni, Cinara largirostris, Macrosiphoniella kuwayamai, Trichosiphonaphis lonicerae* | Shouning, Wuyishan |
| *Iridomyrmex anceps* | *Aphis aurantii, Aphis odinae, Aphis spiraecola, Chaitophorus saliniger, Melanaphis donacis, Periphyllus koelreuteriae* | Fuzhou |
| *Lasius niger* | *Aphis gossypii* | Wuyishan |
| *Lepisiota rothneyi* | *Greenidea ficicola, Aphis spiraecola* | Quanzhou |
| *Liometopum sinense* | *Aphis spiraecola* | Shouning |
| *Monomorium chinense* | *Aphis gossypii, Aphis kurosawai , Macrosiphoniella kuwayamai* | Shouning, Wuyishan |
| *Monomorium* sp. | *Aphis gossypii* | Fuzhou |
| *Nylanderia bourbonica* | *Aphis aurantii, Aphis craccivora, Aphis gossypii, Aphis odinae, Aphis spiraecola, Chaitoregma tattakana, Hysteroneura setariae* | Fuzhou, Shouning, Wuyishan |
| *Nylanderia pubens* | *Aphis spiraecola* | Shouning |
| *Nylanderia flaviabdominis* | *Aphis fabae solanella, Aphis glycines, Aphis gossypii, Aphis spiraecola, Ovatus crataegarius* | Shouning, Wuyishan |
| *Nylanderia flavipes* | *Aphis aurantii, Aphis odinae, Aphis fabae solanella, Aphis kurosawai, Aphis spiraecola, Semiaphis heraclei* | Fuzhou, Shouning, Wuyishan |
| *Ochetellus glaber* | *Aphis aurantii, Aphis gossypii, Aphis spiraecola, Mollitrichosiphum tenuicorpus* | Fuzhou, Shouning |
| *Pheidole fervida* | *Aphis gossypii* | Wuyishan |
| *Pheidole noda* | *Aphis aurantii, Aphis odinae, Aulacorthum solani, Aphis fabae solanella, Aphis glycines, Aphis gossypii, Aphis spiraecola, Ceratovacuna hoffmanni, Glyphinaphis bambusae, Melanaphis bambusae, Melanaphis japonica, Trichosiphonaphis lonicerae* | Fuzhou, Shouning, Wuyishan |
| *Pheidole smythiesii* | *Aphis gossypii* | Wuyishan |
| *Plagiolepis manczshurica* | *Aphis odinae* | Quanzhou |
| *Polyrhachis dives* | *Aphis aurantii, Aphis craccivora, Aphis gossypii, Aphis odinae, Aphis spiraecola, Astegopteryx bambucifoliae, Cinara pinea, Hysteroneura setariae, Uroleucon erigeronensis* | Fuzhou, Shouning |
| *Polyrhachis illaudata* | *Aphis aurantii, Aphis spiraecola* | Fuzhou, Shouning |
| *Prenolepis emmae* | *Aphis gossypii, Aphis kurosawai, Aphis spiraecola, Trichosiphonaphis lonicerae* | Wuyishan |
| *Pristomyrmex punctatus* | *Aleurodaphis mikaniae, Aphis aurantii, Aphis fabae solanella, Aphis glycines, Aphis gossypii, Aphis kurosawai, Aphis odinae, Aphis spiraecola, Ceratoglyphina bambusae, Hysteroneura setariae, Macrosiphoniella kuwayamai, Matsumuraja rubifoliae, Megoura lespedezae, Melanaphis japonica, Semiaphis heraclei, Trichosiphonaphis lonicerae, Uroleucon monticola,* | Fuzhou, Shouning, Wuyishan |
| *Tapinoma sp3* | *Aphis kurosawai* | Fuzhou |
| *Tapinoma melanocephalum* | *Aphis aurantii, Aphis fabae solanella, Aphis gossypii, Aphis odinae, Aphis spiraecola, Schizaphis scirpi* | Fuzhou |
| *Tapinoma sp1* | *Aphis gossypii* | Fuzhou |
| *Tapinoma sp2* | *Aphis spiraecola* | Shouning |
| *Technomyrmex albipes* | *Aphis aurantii, Aphis fabae solanella, Aphis gossypii, Aphis odinae, Ceratovacuna keduensis,* | Fuzhou, Shouning, Wuyishan |
| *Tetramorium bicarinatum* | *Aphis gossypii, Aphis spiraecola, Mollitrichosiphum tenuicorpus, Myzus persicae, Myzus varians* | Fuzhou, Shouning, Wuyishan |
| *Tetramorium caespitum* | *Aphis glycines, Aphis kurosawai , Aphis spiraecola* | Wuyishan |
| *Tetramorium wroughtonii* | *Aphis odinae* | Wuyishan |
