# Supplementary material for: DNA barcoding of aphid-associated ants (Hymenoptera, Formicidae) in a subtropical area of southern China
Source: Zookeys. 2019 Oct 9;879:117–36. doi: 10.3897/zookeys.879.29705 (PMC6795625; doi:10.3897/zookeys.879.29705)
Supplement: Supplementary material 3 [file zookeys-879-117-s003.docx]

Supplementary Figure 1. Line chart of genetic distances of COI sequences based on p-distance and K2P model at different taxonomic levels.
