# Supplementary figures and images for: DNA barcoding of aphid-associated ants (Hymenoptera, Formicidae) in a subtropical area of southern China
Source: Zookeys. 2019 Oct 9;879:117–36. doi: 10.3897/zookeys.879.29705 (PMC6795625; doi:10.3897/zookeys.879.29705)

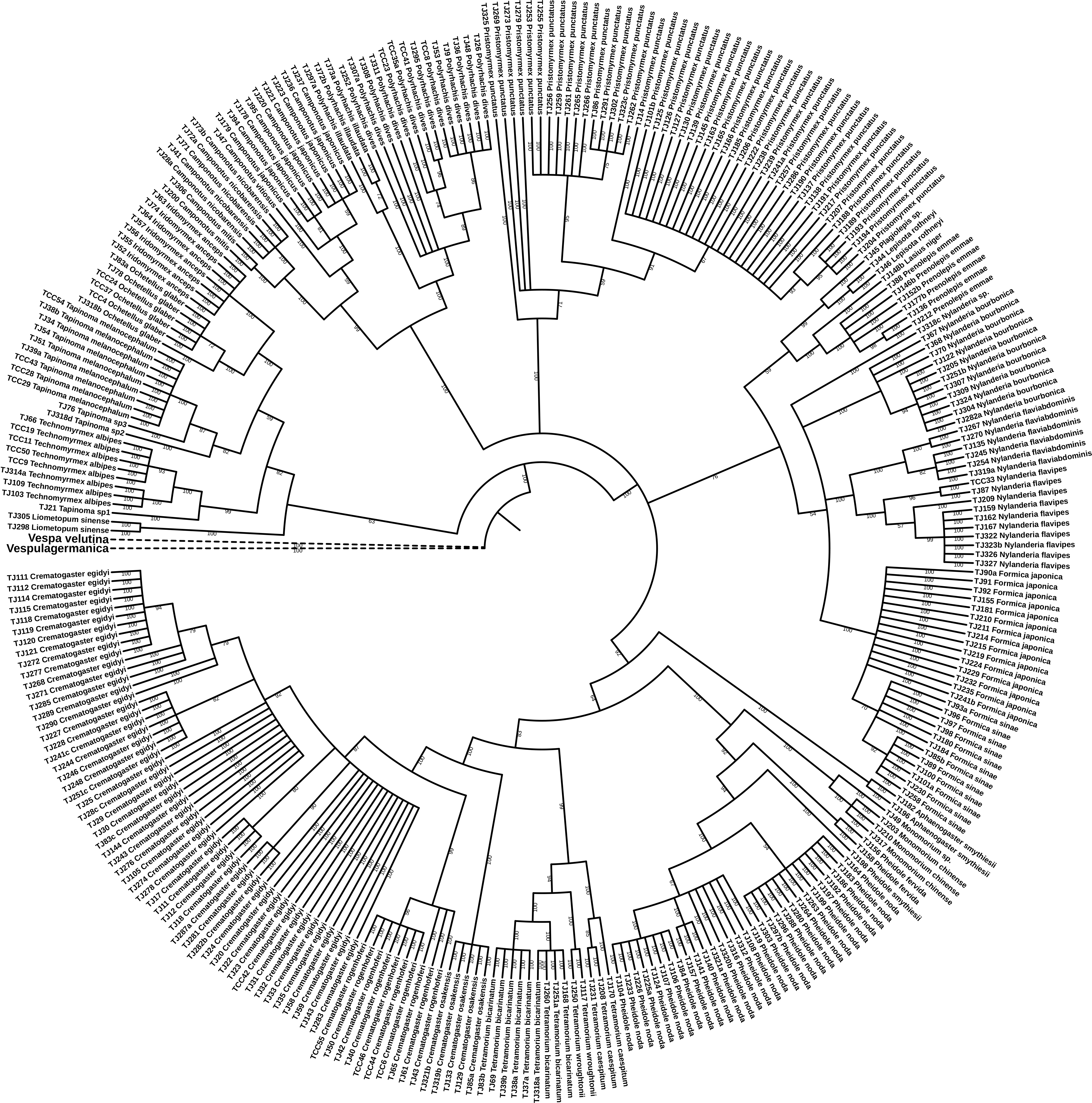

Supplement: Supplementary material 4 [file zookeys-879-117-s004.tif]
